# Supplementary figures and images for: Prognostic nutritional index as a prognostic factor for renal cell carcinoma: A systematic review and meta-analysis
Source: PLoS One. 2022 Aug 5;17(8):e0271821. doi: 10.1371/journal.pone.0271821 (PMC9355260; doi:10.1371/journal.pone.0271821)

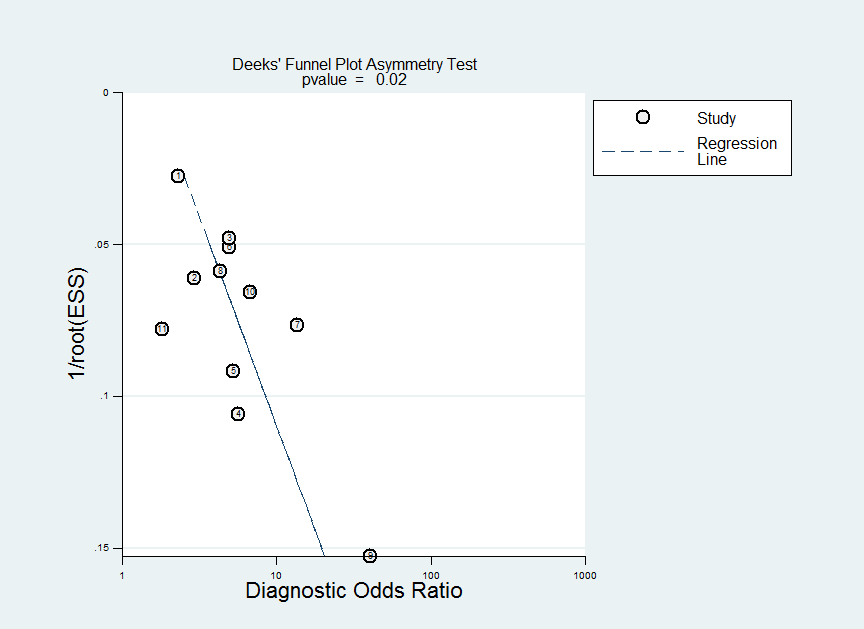

Supplement: S1 Fig — (TIF) [file pone.0271821.s001.tif]
